# Novel AT2 Cell Subpopulations and Diagnostic Biomarkers in IPF: Integrating Machine Learning with Single-Cell Analysis

**DOI:** 10.3390/ijms25147754

**Published:** 2024-07-15

**Authors:** Zhuoying Yang, Yanru Yang, Xin Han, Jiwei Hou

**Affiliations:** Department of Biochemistry and Molecular Biology, School of Medicine, Nanjing University of Chinese Medicine, Nanjing 210023, China; yangzhuoying0102@163.com (Z.Y.); yrr@njucm.edu.cn (Y.Y.)

**Keywords:** IPF, AT2, machine learning, bioinformatics, predictive model

## Abstract

Idiopathic pulmonary fibrosis (IPF) is a long-term condition with an unidentified cause, and currently there are no specific treatment options available. Alveolar epithelial type II cells (AT2) constitute a heterogeneous population crucial for secreting and regenerative functions in the alveolus, essential for maintaining lung homeostasis. However, a comprehensive investigation into their cellular diversity, molecular features, and clinical implications is currently lacking. In this study, we conducted a comprehensive examination of single-cell RNA sequencing data from both normal and fibrotic lung tissues. We analyzed alterations in cellular composition between IPF and normal tissue and investigated differentially expressed genes across each cell population. This analysis revealed the presence of two distinct subpopulations of IPF-related alveolar epithelial type II cells (IR_AT2). Subsequently, three unique gene co-expression modules associated with the IR_AT2 subtype were identified through the use of hdWGCNA. Furthermore, we refined and identified IPF-related AT2-related gene (IARG) signatures using various machine learning algorithms. Our analysis demonstrated a significant association between high IARG scores in IPF patients and shorter survival times (*p*-value < 0.01). Additionally, we observed a negative correlation between the percent predicted diffusing capacity for lung carbon monoxide (% DLCO) and increased IARG scores (cor = −0.44, *p*-value < 0.05). The cross-validation findings demonstrated a high level of accuracy (AUC > 0.85, *p*-value < 0.01) in the prognostication of patients with IPF utilizing the identified IARG signatures. Our study has identified distinct molecular and biological features among AT2 subpopulations, specifically highlighting the unique characteristics of IPF-related AT2 cells. Importantly, our findings underscore the prognostic relevance of specific genes associated with IPF-related AT2 cells, offering valuable insights into the advancement of IPF.

## 1. Introduction

Idiopathic pulmonary fibrosis (IPF) represents a challenging interstitial lung disease that involves the remodeling of alveolar tissue, proliferation of fibroblasts, and accumulation of excess extracellular matrix (ECM) [1,2]. With a median survival rate of approximately three years post-diagnosis and limited treatment options [3,4], it is imperative to comprehensively understand the pathogenesis of IPF and devise effective early detection and treatment approaches.

Epithelial cell dysfunction plays a crucial role in the pathogenesis of IPF [5,6,7]. Alveolar epithelial type II cells (AT2) constitute a diverse and metabolically active population crucial for surfactant production and lung function maintenance [8,9]. The available evidence indicates that injury or dysfunction of AT2 cells plays a crucial role in the pathogenesis of fibrogenesis, leading to the activation of fibroblasts, disruption of lung architecture, and excessive deposition of extracellular matrix (ECM), ultimately resulting in the development of pulmonary fibrosis [10,11,12]. These insights highlight the potential of AT2 cells as both prognostic markers and therapeutic targets.

AT2 cells display functional diversity in lung homeostasis and disease [13,14]. Recent advancements in single-cell RNA sequencing (scRNA-seq) have greatly improved our comprehension of cellular heterogeneity in diverse pathological contexts [15,16]. While previous studies have characterized the transcriptional profiles of alveolar epithelial cells in normal and fibrotic lung tissues through high-throughput analyses [17], a comprehensive understanding of the composition, gene expression patterns, and specific roles of AT2 cell subtypes in fibrotic lung tissue remains elusive. Furthermore, the clinical relevance and prognostic significance of AT2 cell subtypes in fibrosis warrant further investigation. Machine learning, as an automated method for constructing analytical models, holds promise in drug discovery, pathology identification, and model prediction [18,19,20]. Its widespread application in diagnosing and treating various diseases underscores its unique advantages.

**Figure 1:**
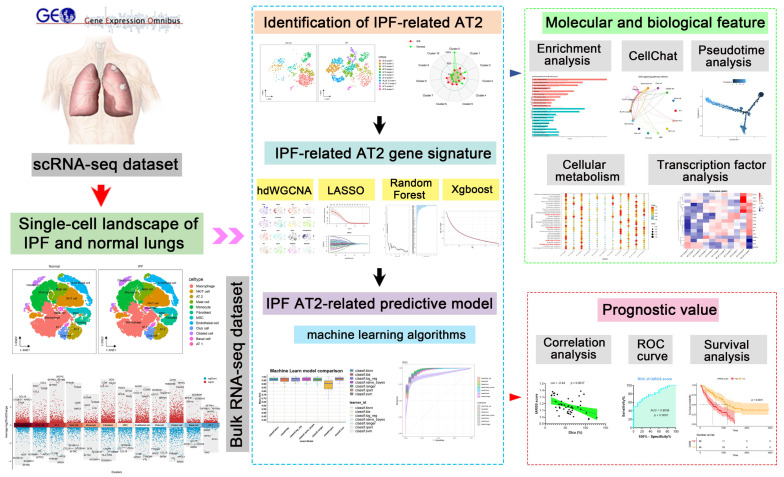
Schematic illustration of our analytic approaches. AT2 cell populations were methodically characterized utilizing publicly accessible single-cell RNA sequencing data, elucidating their molecular and biological features within fibrotic lung tissue. High-dimensional weighted gene co-expression network analysis (hdWGCNA) confirmed co-expression gene modules unique to IPF-AT2, revealing novel gene networks associated with pulmonary fibrosis. Machine learning methodologies were utilized to investigate the prognostic capabilities of these IPF-related subtype gene signatures.

In this study, we systematically classified the AT2 cell population using publicly available scRNA-seq data and elucidated the molecular and biological characteristics of AT2 subtypes in fibrotic lung tissue. Through high-dimensional weighted gene co-expression network analysis (hdWGCNA), we validated co-expression gene modules associated with IPF-AT2 and explored the potential prognostic value of these IPF-related subtype gene signatures using machine learning techniques (Figure 1). Our research introduces several innovative elements: it provides a comprehensive single-cell level classification of AT2 subtypes in IPF, identifies unique molecular and biological features specific to these subtypes, and employs advanced computational approaches to reveal prognostic gene signatures. This integrated approach offers valuable insights into the cellular heterogeneity within AT2 populations and their implications for IPF progression, thereby paving the way for potential targeted therapeutic strategies.

## 2. Results

### 2.1. Single-Cell Transcriptional Landscape of AT2 Cells in Normal and Fibrotic Lung Tissues

**Figure 2:**
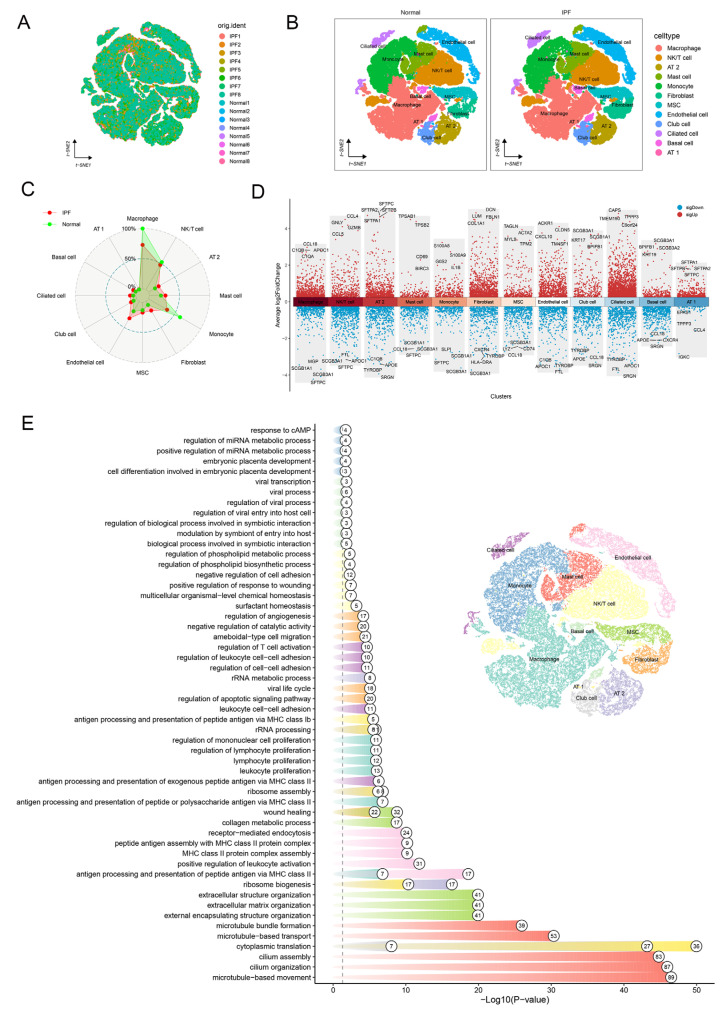
Molecular characterization of the cellular heterogeneity of human IPF lung tissue according to dataset GSE128033. (**A**) t-SNE visualization illustrating cell clusters sorted from normal and IPF lung tissue. (**B**) Cellular populations identified. (**C**) Distribution of different cellular populations in normal and fibrotic lung tissue. (**D**) LogFC visualization for the top six genes in each cell type, showcasing the comparison between normal and fibrotic lung tissue post-differential analysis. (**E**) Representative enriched GO terms enriched in each cell type are depicted. The numbers at the front end of the column represent the number of genes enriched in the GO term.

To investigate the heterogeneity of AT2 cells within human IPF lung tissue, we collected and analyzed scRNA-seq data from IPF patients and normal controls. Following the implementation of quality control procedures and the mitigation of batch effects, a total of 63,251 qualified cells expressing 22,559 genes were obtained. Specifically, IPF patients contributed 34,787 cells expressing 22,237 genes, while healthy controls contributed 28,464 cells expressing 20,475 genes. Following cell annotation, we identified twelve major cell populations, including macrophages, NK/T cells, AT2 cells, mast cells, monocytes, fibroblasts, mesenchymal stem cells (MSCs), endothelial cells, club cells, ciliated cells, basal cells, and AT1 cells (Figure 2A,B). Subsequently, we examined alterations in cellular composition between IPF and normal tissue (Figure 2C). Additionally, we conducted an analysis of differentially expressed genes (DEGs) across each cell population (Figure 2D).

**Figure 3:**
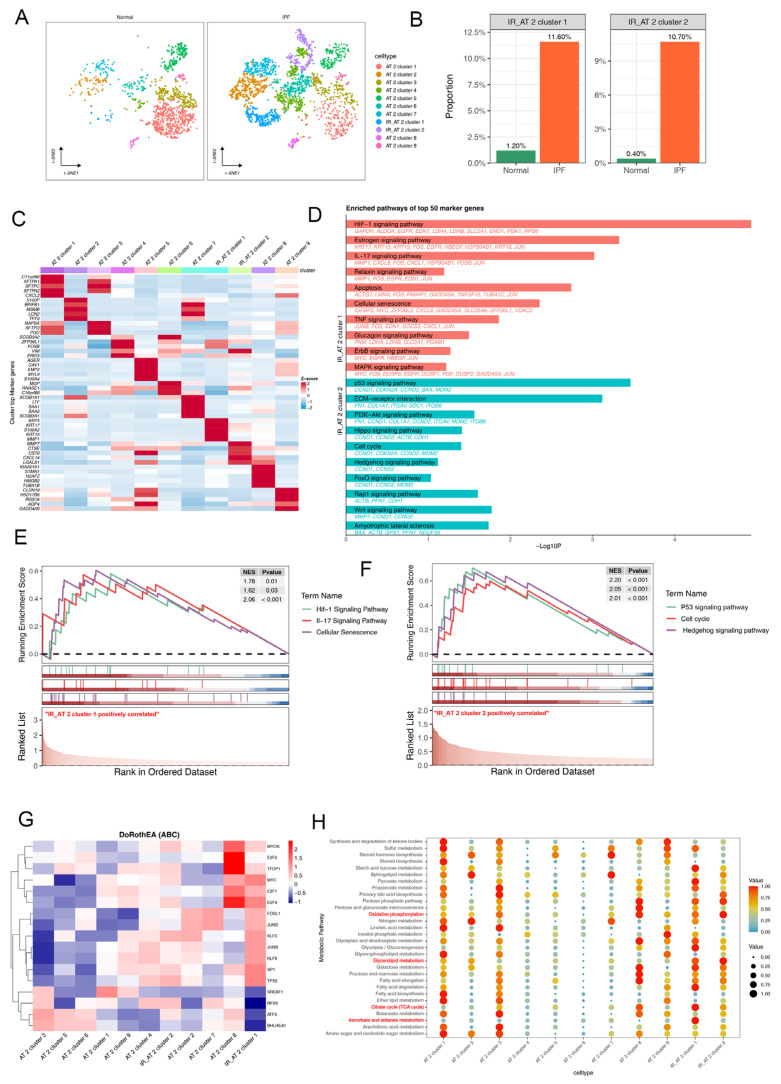
Single-cell analysis reveals the heterogeneity of AT2 cells in IPF. (**A**) 2D visualization of single-cell clustering of AT2 profiles for all AT2 cells in normal and fibrotic lung samples. (**B**) Proportions of IPF-related AT2 subclusters in each group. (**C**) Heatmap exhibit representative differentially expressed genes (DEGs) across each cell population. (**D**) Enriched KEGG pathways of upregulated DEGs in IPF-related AT2 subclusters. (**E**,**F**) GSEA enrichment plots display representative signaling pathways that are upregulated in IR_AT2 cluster compared to other AT2 cell subtypes. (**G**) Heatmap displaying transcription factor analysis scores in the eleven AT2 cell subclusters. (**H**) Dot plot showing the activation status of metabolic pathways from each of the eleven AT2 cell subclusters. IR_AT2, IPF-related AT2.

We then repeated t-distributed stochastic neighbor embedding (t-SNE) analysis to hierarchically cluster the AT2 cells. Following cell annotation, we classified the AT2 cells into 11 putative cell populations (Figure 3A). Importantly, we demonstrated that clusters 10 and 11 of AT2 cells were significantly increased in fibrotic lung tissue compared to normal lung tissues, defining these as IPF-related AT2 (IR_AT2) clusters (Figure 3A,B, and Figure S1). This finding suggests a potential pivotal role for these specific cell populations in fibrogenesis. The expression of cell-subtype-specific marker genes in AT2 cells was detected and presented in a heatmap (Figure 3C).

To further investigate the potential molecular mechanisms of IPF-related AT2 cell involvement in the development of pulmonary fibrosis, we conducted functional enrichment analysis of highly expressed characteristic genes in IPF-related AT2 cells. The results demonstrated significant associations between the HIF1 and IL17 signaling pathways, cellular senescence, and IR_AT2 cluster 1, while characteristic genes of IR_AT2 cluster 2 were notably enriched in the p53 and hedgehog signaling pathways, and the cell cycle (Figure 3D–F). Furthermore, transcription factor analysis revealed significant activation of the transcription factors *KLF5* and *MYC* within IR_AT2 cluster 1 and IR_AT2 cluster 2, respectively (Figure 3G).

A large body of research suggests a close association between metabolic abnormalities and the progression of IPF [3,4]. Therefore, we analyzed the alterations in various metabolic processes within different subtypes of AT2 cells. The results indicated a significant enhancement of ascorbate and aldarate metabolism and glycerolipid metabolism within the IR_AT2 compared to other cell subpopulations (Figure 3F). These findings suggest that IR_AT2 plays a crucial role in modulating energy metabolism, indicating their potential involvement in the onset and advancement of IPF.

### 2.2. Pseudo-Time Trajectory Analysis and Cell Communication Analysis

**Figure 4:**
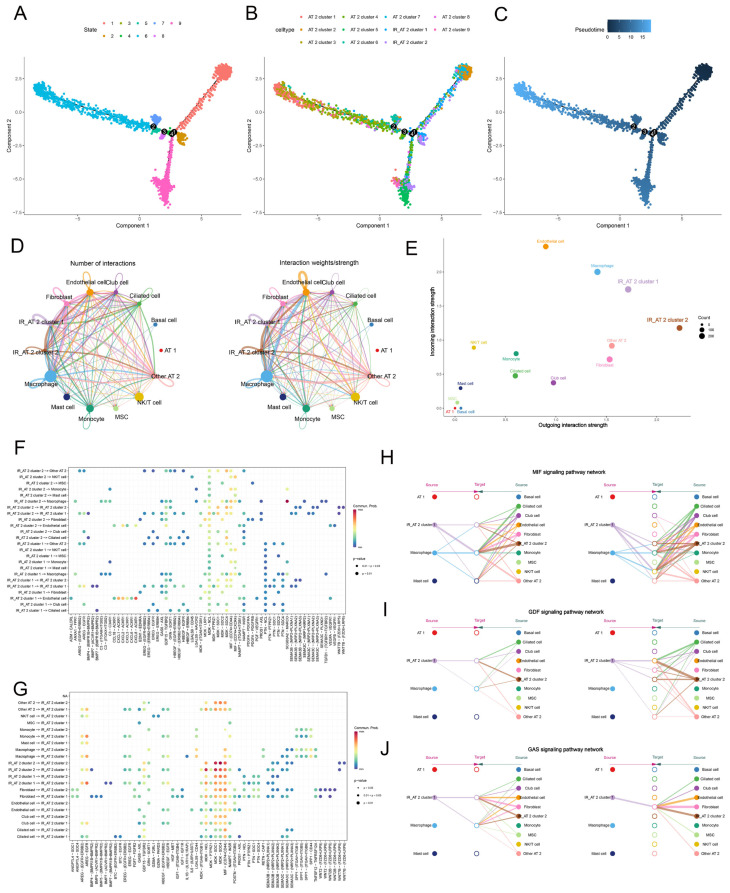
Trajectory and cell–cell communication analysis of AT2 cell populations in IPF patients. (**A**–**C**) The developmental trajectory analysis of AT2 cells, colored by states (**A**), cellular subpopulations (**B**), and pseudotime (**C**). (**D**–**J**) Cell–cell communication analysis in AT2 cell subpopulations. (**D**) Circle plots depict the number and strength of ligand–receptor interactions between pairs of cell populations. (**E**) A scatter plot reveals the variations in incoming and outgoing interaction strengths across all cell types. (**F**,**G**) Networks depicting cell–cell ligand–receptor (LR) and cytokine-related pathways show how IR_AT2 cluster 1 and IR_AT2 cluster 2 interact with other cell populations. (**H**–**J**) Hierarchical plots depict the inferred intercellular communication networks for MIF (**H**), GDF (**I**), and GAS signaling (**J**).

To investigate the origins of IR_AT2 cells in the development of IPF, we performed pseudo-time trajectory analysis of AT2 cells. This analysis revealed the unique position of IR_AT2 cells on the developmental trajectory (Figure 4A–C). The emergence of the IR_AT2 cluster was noted during the intermediate stages of the trajectory. Specifically, the IR_AT2 cluster was identified at trajectory branches 1 and 4, indicating a distinct developmental pathway for these cells (Figure 4A–C).

To systematically examine the cell–cell interactions of various AT2 cell subpopulations, we utilized CellChat, which leverages known ligand–receptor (L-R) pairs and their cofactors. Our analysis revealed that IR_AT2 cells exhibited robust communication abilities with other cell subtypes involved in IPF pathogenesis (Figure 4D). Both IR_AT2 cluster 1 and IR_AT2 cluster 2 demonstrated stronger secretory capacities, as indicated by their higher outgoing interaction strengths compared to other cell populations (Figure 4E). Notably, our study found that IR_AT2 clusters can directly interact with other AT2 cell subtypes through adhesive ligand–receptor pairs such as MIF/CD74/CD44, GDF5/TGFBR2, and GAS6/AXL (Figure 4F,G). Furthermore, our CellChat analysis indicated an upregulation of pro-inflammatory signaling pathways (including MIF, GDF, and GAS) in the communication between IR_AT2 cells and other cell subtypes (Figure 4H–J and Figure S2).

### 2.3. Identification of the Crucial Modules Related to IPF-Related AT2 by hdWGCNA

**Figure 5:**
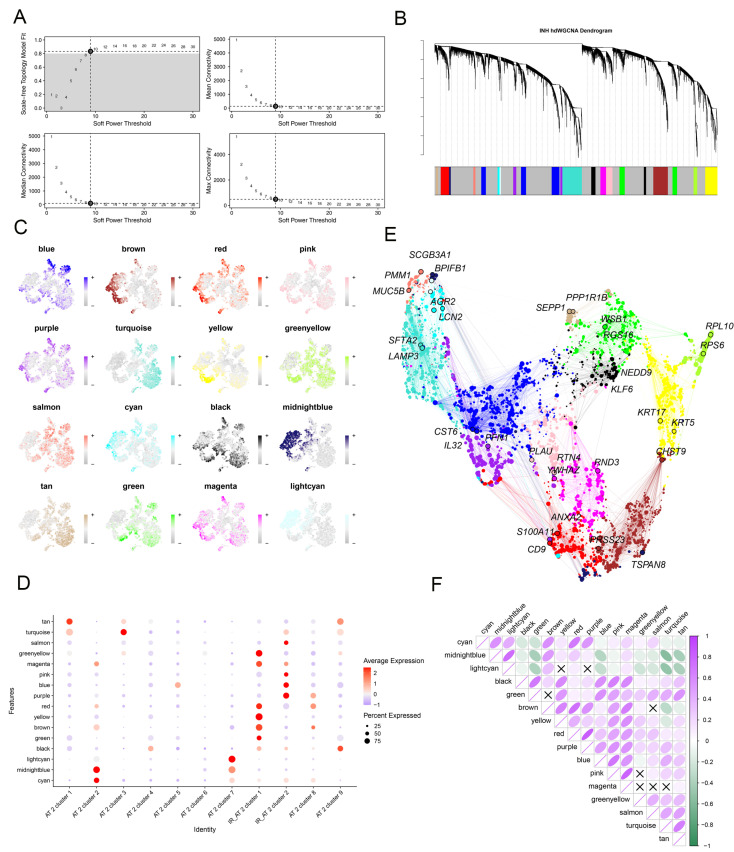
hdWGCNA identify the hub modules associated with IPF-related AT2 cell. (**A**) Construction of a scale-free network using a soft-threshold power of 9. (**B**) Classification of highly variable genes into 12 modules through hdWGCNA. (**C**) t-SNE plots derived from scRNA-seq data (similar to Figure 2A), with colors representing module eigengenes (MEs) for the identified co-expression gene modules. (**D**) Assessment of module activity across different AT2 cell clusters using hdWGCNA. (**E**) A UMAP diagram illustrates the co-expression network across eight gene modules. Node sizes correspond to kME values, and nodes are colored based on their co-expression module assignment. The top two hub genes per module are annotated. (**F**) The matrix plot visually depicts inter-module relationships by illustrating correlations between module eigengenes.

We utilized high-dimensional weighted gene co-expression network analysis (hdWGCNA), an extensive framework for co-expression network analysis in scRNA-seq data, to examine key modules within IR_AT2 cells. Using an optimal soft-thresholding power of 9, we established a scale-free co-expression network (Figure 5A). This analysis revealed twelve distinct gene co-expression modules (Figure 5B,C and Figure S3). Notably, the yellow, green-yellow, blue, and purple modules exhibited high levels of activation predominantly within IR_AT2 cells (Figure 5D). Furthermore, we explored the correlations among the modules (Figure 5E,F).

### 2.4. Various Machine Learning Algorithms Identifies Signature Genes for IPF-Related AT2 Cell

**Figure 6:**
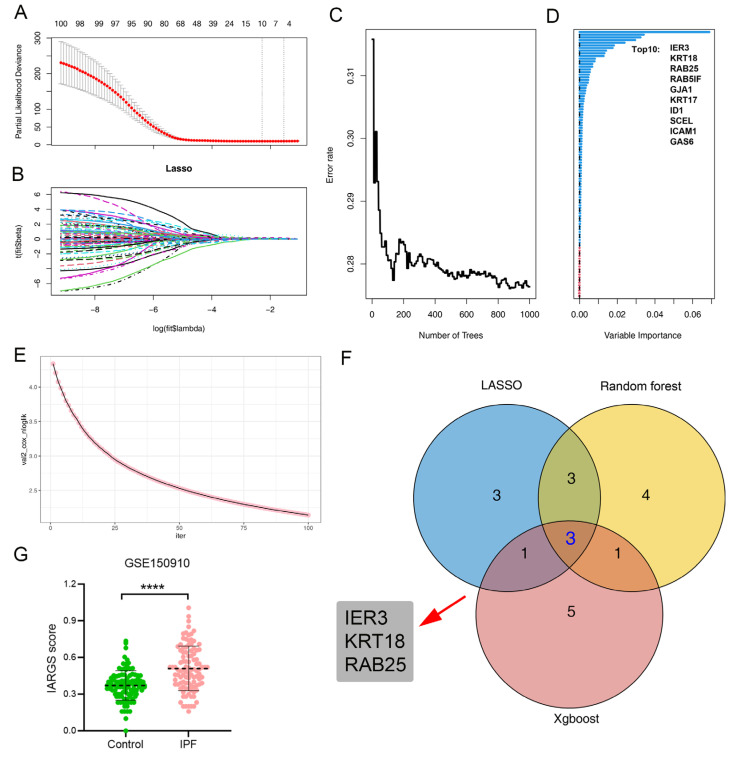
Screening IR_AT2-related hub genes by machine learning. (**A**,**B**) Application of the least absolute shrinkage and selection operator (LASSO) regression algorithm. (**A**) Feature selection using the LASSO algorithm. (**B**) Coefficient changes observed in the selected features using the LASSO algorithm. (**C**,**D**) Ranking of all genes based on their importance in the model using random forest analysis. (**C**) Gene ranking using random forest analysis. (**D**) Assessment of feature importance through random forest analysis. (**E**) Variation in the negative log partial likelihood of Cox proportional hazards regression with the XGBoost algorithm across different iterations. (**F**) A Venn diagram illustrating the genes identified as overlapping among the three algorithms. (**G**) Boxplots presenting the levels of IPF-related AT2 genes (IARGs) scores in lung tissue from IPF patients (n = 103) and normal controls (n = 103). Results are depicted as means ± SD (**** *p* < 0.0001).

Based on the hdWGCNA screening results, we further employed three machine learning algorithms to identify hub genes associated with IPF-related AT2 cells, using an external dataset (GSE70866). The LASSO regression algorithm identified 10 key genes significantly associated with the prognosis of IPF patients (Figure 6A,B). Random forest analysis ranked all genes by importance and highlighted the top 30 (Figure 6C,D). In addition, the Xgboost algorithm filtered out 10 critical genes (Figure 6E). We then used a Venn diagram to visualize overlapping genes from the three machine learning methods (Figure 6F), identifying three crucial genes: *IER3*, *KRT18*, and *RAB25*, all of which are closely correlated with the prognosis of IPF patients.

### 2.5. Machine Learning-Based Construction of the Predictive Model for IPF

To gain a deeper understanding of the correlation between the gene signature of IR_AT2 cells and fibrogenesis, we began by analyzing the scores of genes associated with IPF-related AT2 cells (IARG) in both normal and fibrotic lung tissues. The findings showed a significant increase in IARG scores in IPF lung tissue, suggesting a potential correlation between these genes and the fibrogenesis process (Figure 6G).

**Figure 7:**
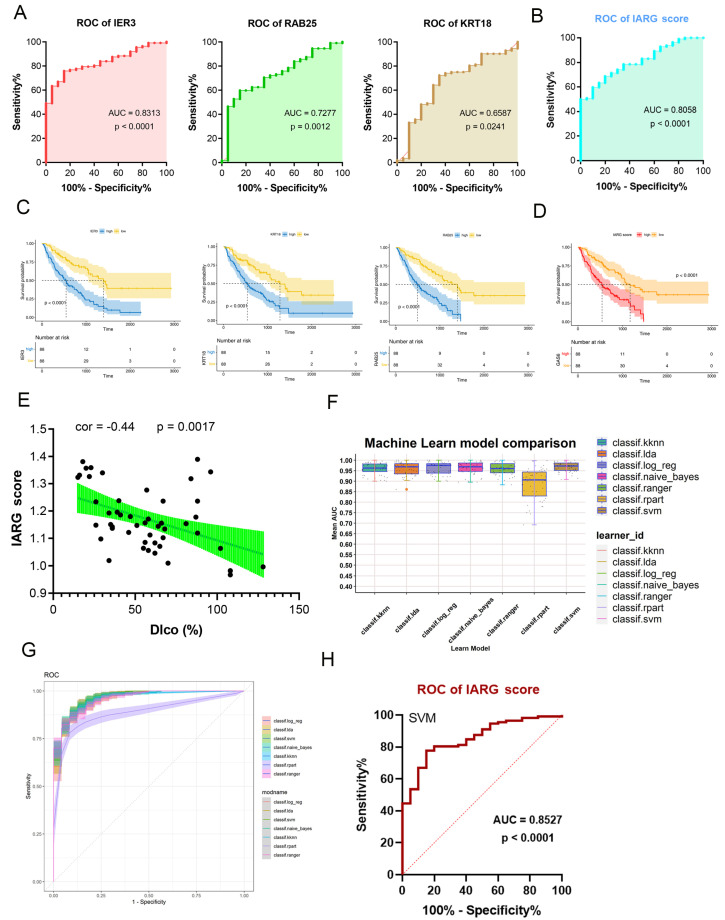
Application of machine learning for predictive modeling. (**A**,**B**) Analysis of receiver operating characteristic (ROC) curves for identified hub genes (**A**) and the IARG score related to IPF-AT2 cells (**B**). (**C**,**D**) Kaplan–Meier survival analysis conducted on IPF patients based on gene expression patterns (**C**) and the IARG score (**D**). (**E**) Examination of the correlation between the IARG score and diffusion lung capacity for CO (% DLCO) in both control and IPF patients. (**F**) Development of predictive models utilizing seven different machine learning algorithms. (**G**) Display of ROC values from 10 repetitions of 5-fold cross-validation across all seven algorithms in the training cohort. (**H**) Presentation of the ROC curve specifically for the “svm” machine learning algorithm model in the validation cohort.

We then turned our attention to predicting the onset and progression of IPF using a predictive model built on the identified IARG signature. Two bulk RNAseq datasets were utilized for further analysis. Initially, we conducted receiver operating characteristic (ROC) curve analysis to assess the diagnostic performance of the identified hub genes. The results revealed that *IER3*, *KRT18*, *RAB25*, and the overall IARG score all exhibited AUC values above 0.65, indicating that this three-gene signature effectively distinguishes IPF patients (Figure 7A,B). Furthermore, elevated levels of these three genes and a high IARG score in patients with IPF were associated with shorter survival times (Figure 7C,D). Consistently, there is a negative correlation (correlation coefficient = −0.44, *p*-value < 0.05) between the percent predicted diffusing capacity for lung carbon monoxide (% DLCO) and the increased IARG score, as shown in Figure 7E.

The final step consisted of applying seven machine learning algorithms and optimizing parameters for each model, following five repetitions of tenfold cross-validation. Using the GSE110147 dataset for training, and the GSE32537 dataset for evaluating the final model’s predictive power, we evaluated the AUC values for both models. The “svm” machine learning algorithm model was ultimately selected due to its superior performance metrics, including a high AUC of 0.85 and a statistically significant *p*-value of less than 0.0001, as illustrated in Figure 7G,H.

## 3. Discussion

AT2 cells are a heterogeneous population that have emerged as a central part of idiopathic pulmonary fibrosis (IPF) pathophysiology [5,21]. The molecular characteristics and clinical relevance of AT2 heterogeneity in IPF remain unknown, despite decades of investigation. In this study, we meticulously analyzed the AT2 cell landscape in IPF lung tissue, identifying two predominant subpopulations primarily found in fibrotic lung tissues, termed IPF-related AT2 (IR_AT2). Subsequently, we conducted a detailed examination of the unique characteristics and clinical relevance of these various AT2 subtypes. This meticulous investigation not only provides essential insights for a more thorough understanding of the pathogenesis of IPF but also presents significant evidence supporting the prognostic value linked to IR_AT2-specific gene signatures.

A few previous studies have investigated the heterogeneity of AT2 populations in pulmonary fibrosis [17,22], offering valuable insights but only providing a snapshot of the overall picture. Our thorough characterization of AT2 heterogeneity led to the discovery of two IPF-related subtypes: IR_AT2. IR_AT2 exhibited increased expression of *MYC* and *CDKN2A*, implying that cellular senescence and the cell cycle may be essential for the transdifferentiation of this profibrotic AT2 phenotype. Interestingly, we noted that IR_AT2 also expressed high levels of *S100A2* and *MMP7* soluble factors, suggesting that this AT2 subset could play a role in establishing a critical profibrotic microenvironment necessary for (myo)fibroblast differentiation [23,24]. In the future, additional research is necessary to clarify the roles of *S100A2* and *MMP7* in IR_AT2 cells, their potential interplay with the immune system, and their involvement in fibrogenesis. Another recent study has identified a novel population of AT2 cells that are enriched in PD-L1 expression and expand following pneumonectomy [25]. This suggests that AT2 cells exhibit varying levels of heterogeneity in different pathological and physiological processes. Furthermore, theoretical models and simulations, as discussed in a recent study [26], have begun to explore the heterogeneities present within colonies, offering valuable insights for the theoretical examination of cell heterogeneity. In forthcoming research endeavors, the development of dual lineage tracing methodologies reliant upon the expression patterns of *S100A2* and *MMP7* will be crucial for the precise labeling of IR_AT2 cells. Importantly, our results revealed that IR_AT2 displayed heightened expression of genes linked to profibrotic signaling pathways, including IL17 and hedgehog signaling pathways [27,28], indicating the significant molecular pathways that IR_AT2 may influence in the profibrotic microenvironment. 

AT2 cells are recognized for their crucial secretory function in the alveolus, contributing to lung homeostasis maintenance [8,29,30]. ScRNA-seq could provide insights into how IR_AT2 and other cells communicate in fibrotic lung tissue. Cell–cell communication analysis revealed that interactions between IR_AT2 and fibroblasts may be mediated by ligand receptors such as MIF/CD74/CD44 and GDF15/TGFBR2. Furthermore, we identified fibrosis-associated signaling pathways, including MIF and GAS signaling pathways [31,32], which were found to be upregulated in lung fibrosis. Our pseudo-temporal analysis and trajectory study of IR_AT2 revealed that the majority of IR_AT2 are mainly clustered in the intermediate stages of AT2 differentiation, suggesting that IR_AT2 may originate from the activation of resident normal AT2 cells. Further studies to investigate the origin of these IR_AT2 subpopulations using transgenic mice and lineage tracing techniques are required. As a result of these findings, it may be possible to target the identified IR_AT2 subpopulation for treatment of fibrosis.

A prognostic gene signature related to IR_AT2 was established by utilizing hdWGCNA, an advanced bioinformatics strategy [33], LASSO analysis, random forest, and Xgboost analysis. As a consequence, three genes were identified as strongly related to IPF prognosis—*IER3*, *KRT18*, and *RAB25*. While previous studies have indicated that certain proteins, including RAB25, may stimulate the activation of hepatic stellate cells in liver fibrosis [34], the involvement of these factors in pulmonary fibrogenesis remains largely unknown and necessitates further exploration. 

The IPF-AT2-related gene (IARG) scores were then utilized to develop a predictive model for IPF related to IR_AT2. Following multiple validations, the SVM model emerged as the top choice due to its superior accuracy, sensitivity, and specificity. Further validation of the model’s accuracy in independent cohorts confirms its relevance to disease prediction and treatment.

While these data are important for advancing knowledge, we need to acknowledge a few limitations. First, given the computational and omic nature of this work, it is imperative to validate the IR-AT2-related genes identified in our findings in order to identify and characterize IR-AT2 subtypes present in fibrotic lung tissues using experimental methods. Moving forward, we intend to conduct pertinent experiments to validate the aforementioned results and delve deeper into the regulatory mechanisms and clinical significance of the new subgroups of AT2. Second, our study is constrained by the absence of a large clinical cohort, which could have further enhanced and refined our analytical results.

## 4. Materials and Methods

### 4.1. Data Acquisition and Processing

Five independent public datasets were downloaded from the NCBI GEO databases (http://www.ncbi.nlm.nih.gov/geo/, accessed on 2 February 2024). Figure 1 illustrates the flowchart of this study. Specifically, we utilized the scRNA-seq dataset GSE128033 to explore the heterogeneity of AT2 cell populations in both normal and IPF lung tissues [35]. Data processing for scRNA-seq was conducted using the R package “Seurat” (version: 4.3.0), following established protocols [36]. Cells with gene expression levels below 300 genes or above 6500 genes, and those with mitochondrial gene expression exceeding 10%, were excluded from the analysis, ensuring the inclusion of the majority of cells in the datasets. We applied the SCTransform function for normalization and scaling of raw counts, followed by principal component analysis (PCA). To mitigate batch effects across dissociated scRNA-seq raw data, we utilized the R package “Harmony” (version: 0.1.1). Through unsupervised cluster analysis and t-distributed stochastic neighbor embedding (t-SNE), discrete cell clusters were identified within each scRNA-seq dataset. After annotating each cell cluster based on known cell type marker genes, we used the “scMetabolism” package (version 0.2.1) to analyze the metabolic activity of each cell type.

In order to develop a predictive model related to IR-AT2 cells using machine learning techniques, two transcriptome sequencing datasets, GSE32537 and GSE110147, were systematically gathered and employed as the training and testing cohorts, respectively. The sample characteristics of all five datasets are summarized in Table 1. An ethics committee approval was not required for analyses of data from a public database.

### 4.2. Trajectory and Cell–Cell Communication Analysis

A pseudo-time trajectory of macrophages was inferred using the DDR-Tree algorithm from the “Monocle” package (version 2.26.0). Using the default settings of the recommended pipelines, the “CellChat” package (version 1.6.1) was utilized for identifying interactions between IR_AT2 and other cell populations [37].

### 4.3. Enrichment Analysis 

Using Seurat’s “FindMarkers” function, each cell sub-cluster’s DEGs were identified. The “clusterProfiler” package (version 4.7.1003) calculated gene set enrichment analysis (GSEA). Using the “GseaVis” package (version 0.0.8), we visualized the functional enrichment result.

### 4.4. High Dimensional Weighted Gene Co-Expression Network Analysis (hdWGCNA)

As mentioned in https://smorabit.github.io/hdWGCNA/articles/basic_t.html, accessed on 10 February 2024 [38], the “hdWGCNA” package (version 0.1.1.90010) was used to identify potential AT2 cell-related genes associated with IPF. Briefly, separately for each sample and cell cluster, metacells were constructed using the hdWGCNA function MetacellsByGroups, aggregating 50 cells per metacell. Briefly, metacells were constructed using the hdWGCNA function MetacellsByGroups, aggregating 50 cells per metacell for each sample and cell cluster. The Seurat object was subsetted initially for the specific cell population of interest, after which the standard hdWGCNA pipeline was applied. This included executing functions such as TestSoftPowers, ConstructNetwork, ModuleEigengenes, ModuleConnectivity, and RunModuleUMAP sequentially with default parameters.

### 4.5. Construction of Machine Learning Model

Further identification of the hub genes was achieved using three machine learning algorithms (random forest, LASSO, and xgboost). In order to determine gene importance, we used the “randomForest” and “caret” packages (version: 4.7-1.1/6.0-94). LASSO regression analyses were carried out using the “ezcox” and “glmnet” packages (version 1.0.4/4.1-7). The xgboost analysis was carried out using the “xgboost” package (version: 1.7.5.1). The predictive model was built using the intersection of three machine-learning-selected genes. The construction of the machine learning models was carried out using the “mlr3” package (version 0.16.0) in R, which encompassed a range of algorithms including logistic regression (log_reg), linear discriminant analysis (LDA), random forest (ranger), support vector machine (SVM), naive Bayes classifier (naive_bayes), recursive partitioning and regression trees (part), and k-nearest neighbors (kknn). ROC curves were generated to assess the accuracy of each model, employing the “timeROC” R package (version 0.4). Afterward, each model’s predictive capability was evaluated, and a final model’s performance was validated by an independent test.

### 4.6. Statistical Analysis

Statistical analyses and data visualizations were conducted utilizing R software (version 4.2.1). Pearson’s correlation coefficients were employed to evaluate the associations between two continuous variables. In the case of quantitative data, either a two-tailed, unpaired Student t-test or a one-way analysis of variance (ANOVA) with Tukey’s multiple comparisons test was utilized to compare values across subgroups. A significance level of *p* < 0.05 was deemed statistically significant.

## 5. Conclusions

Our study enhances our understanding of the heterogeneity present in the AT2 population within fibrotic lung tissue. By uncovering distinctive molecular and biological traits among AT2 subpopulations and highlighting the prognostic importance of signature genes, we contribute valuable insights into IPF development. This research establishes a foundation for future investigations aimed at delineating cellular diversity within fibrotic tissue and exploring its functional and clinical implications. The novelty of our study lies in its comprehensive single-cell level classification of AT2 subtypes in IPF, identification of unique molecular and biological features specific to these subtypes, and use of advanced computational approaches to reveal prognostic gene signatures. This integrated approach offers a deeper understanding of AT2 cellular heterogeneity and its role in IPF progression, paving the way for potential targeted therapeutic strategies.

## Figures and Tables

**Table 1:** A summary of the datasets analyzed.

Dataset	Year	Area	Species	Platform	Data Type	Number of Samples	
						Normal	IPF
GSE128033	2019	USA	*Homo*	GPL18573	scRNA-seq	10	8
GSE150910	2020	USA	*Homo*	GPL24676	Bulk RNA-seq	103	103
GSE32537	2011	USA	*Homo*	GPL6244	Bulk RNA-seq	39	131
GSE110147	2018	Canada	*Homo*	GPL6244	Bulk RNA-seq	11	22
GSE70866	2015	Germany	*Homo*	GPL14550	Bulk RNA-seq	20	212

## Data Availability

The data that support this study are available within the article and its Supplementary Data Files or available from the authors upon request.

## Supplementary Materials

The following supporting information can be downloaded at: https://www.mdpi.com/article/10.3390/ijms25147754/s1.
