# Supplementary material for: Novel AT2 Cell Subpopulations and Diagnostic Biomarkers in IPF: Integrating Machine Learning with Single-Cell Analysis
Source: Int J Mol Sci. 2024 Jul 15;25(14):7754. doi: 10.3390/ijms25147754 (PMC11277372; doi:10.3390/ijms25147754)
Supplement: Supplementary file 1 [file ijms-25-07754-s001.zip › ijms-3077866-supplementary.pdf]

Novel AT2 cell subpopulations and diagnostic biomarkers in IPF: integrating machine learning with single-cell analysis

Zhuoying Yang[1], Yanru Yang[1], Xin Han[1*], Jiwei Hou[1,*]

[1] Department of Biochemistry and Molecular Biology, School of Medicine, Nanjing University of Chinese Medicine, Nanjing, 210023, China.

[*]**Corresponding authors:** Xin Han (xhan0220@njucm.edu.cn)
Jiwei Hou (houjw@njucm.edu.cn)

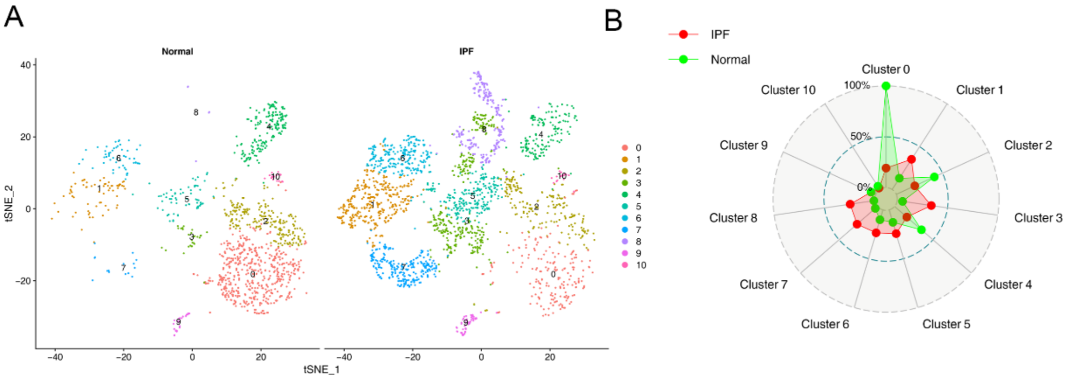

Figure S1：Fibrotic and normal lung AT2 subcluster into distinct cell populations. (A) Subclustering of fibrotic and normal lung fibroblasts further identified 11 distinct subtypes. Color-coded UMAP plot is shown and each fibroblast subcluster is defined on the right. (B) Cell proportions of AT2 subclusters in fibrotic and normal lung tissues. Cells of cluster 6 and cluster 8 were significantly increased in fibrotic samples compared to normal scar samples.

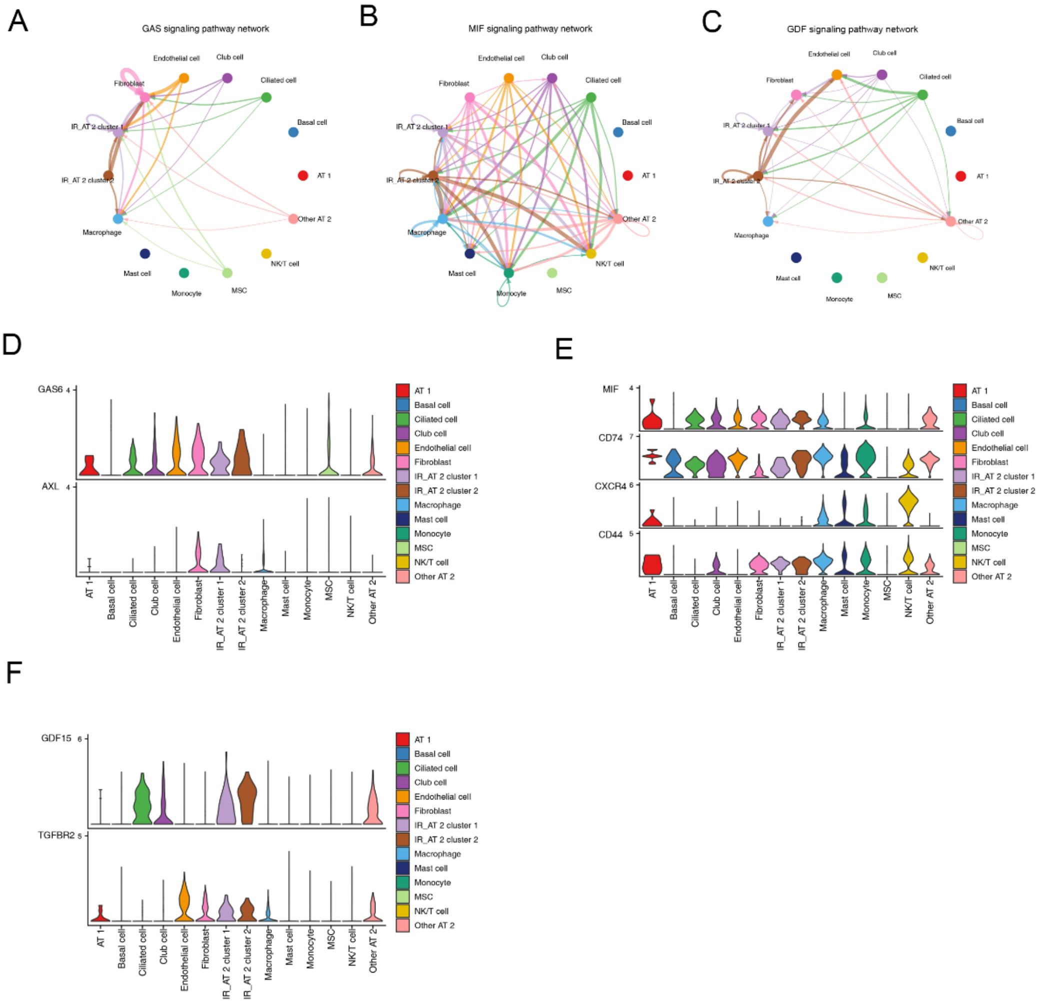

Figure S2：Cell communication analysis in AT2 subpopulations. (A) (A-C) Circle plots showing selected inferred differential signaling networks. The edge width rep-resents the communication probability. (D-F) The violin plot displays the expression of the featured genes in GAS (D), MIF (E), and GDF signaling (F) across all cell subtypes.

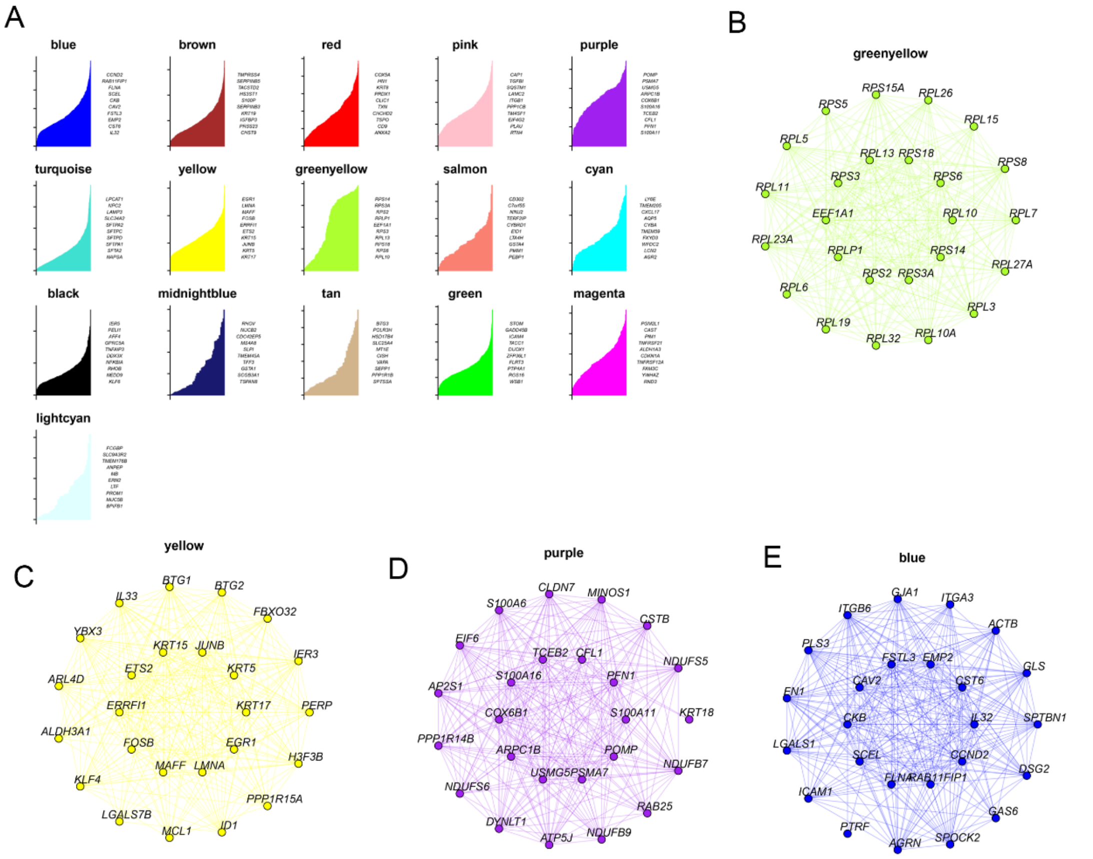

Figure S3: High dimensional weighted gene co-expression network analysis (hdWGCNA) reveals module-specific hub genes in IPF-related fibroblasts. (A) kME (Eigengene-based connectivity) map showing the top 10 hub genes in each module ranked by kME across the macrophages. Genes with higher connectivity or kME values are considered more central or influential within their respective modules. (B-E) Protein–protein interaction (PPI) network of the identified hub genes in each module.
